# Spatial overlap of gray wolves and ungulate prey changes seasonally corresponding to prey migration

**DOI:** 10.1186/s40462-024-00466-w

**Published:** 2024-04-26

**Authors:** Nathaniel H. Wehr, Seth A. Moore, Edmund J. Isaac, Kenneth F. Kellner, Joshua J. Millspaugh, Jerrold L. Belant

**Affiliations:** 1 Department of Fisheries and Wildlife, Michigan State University, East Lansing, MI USA; 2 Department of Biology and Environment, Grand Portage Band of Lake Superior Chippewa, Grand Portage, MN USA; 3 Wildlife Biology Program, Department of Ecosystem and Conservation Sciences, W.A. Franke College of Forestry and Conservation, University of Montana, Missoula, MT USA; 4 Camp Fire Program in Wildlife Conservation, State University of New York College of Environmental Science and Forestry, Syracuse, NY USA

**Keywords:** Brownian bridge movement model, *Canis lupus*, Corridor, Migration Mapper, Migratory coupling, Moose, Predator–prey, Range shift, Space use, White-tailed deer

## Abstract

**Background:**

Prey are more vulnerable during migration due to decreased familiarity with their surroundings and spatially concentrated movements. Predators may respond to increased prey vulnerability by shifting their ranges to match prey. Moose (*Alces alces*) and white-tailed deer (*Odocoileus virginianus*) are primary gray wolf (*Canis lupus*) prey and important subsistence species for Indigenous communities. We hypothesized wolves would increase use of ungulate migration corridors during migrations and predicted wolf distributions would overlap primary available prey.

**Methods:**

We examined seasonal gray wolf, moose, and white-tailed deer movements on and near the Grand Portage Indian Reservation, Minnesota, USA. We analyzed GPS collar data during 2012–2021 using Brownian bridge movement models (BBMM) in Migration Mapper and mechanistic range shift analysis (MRSA) to estimate individual- and population-level occurrence distributions and determine the status and timing of range shifts. We estimated proportional overlap of wolf distributions with moose and deer distributions and tested for differences among seasons, prey populations, and wolf sex and pack affiliations.

**Results:**

We identified a single migration corridor through which white-tailed deer synchronously departed in April and returned in October–November. Gray wolf distributions overlapped the deer migration corridor similarly year-round, but wolves altered within-range distributions seasonally corresponding to prey distributions. Seasonal wolf distributions had the greatest overlap with deer during fall migration (10 October–28 November) and greatest overlap with moose during summer (3 May–9 October).

**Conclusions:**

Gray wolves did not increase their use of the white-tailed deer migration corridor but altered distributions within their territories in response to seasonal prey distributions. Greater overlap of wolves and white-tailed deer in fall may be due to greater predation success facilitated by asynchronous deer migration movements. Greater summer overlap between wolves and moose may be linked to moose calf vulnerability, American beaver (*Castor canadensis*) co-occurrence, and reduced deer abundance associated with migration. Our results suggest increases in predation pressure on deer in fall and moose in summer, which can inform Indigenous conservation efforts. We observed seasonal plasticity of wolf distributions suggestive of prey switching; that wolves did not exhibit migratory coupling was likely due to spatial constraints resulting from territoriality.

**Supplementary Information:**

The online version contains supplementary material available at 10.1186/s40462-024-00466-w.

## Background

### Recognizing place

The Grand Portage Band of Lake Superior Chippewa is a federally recognized sovereign nation of Anishinaabe people with jurisdiction over the Grand Portage Indian Reservation (Gichi Onigaming), Minnesota, USA. The Grand Portage Band exercises its usufructuary rights to food sovereignty through subsistence hunting, fishing, and gathering throughout the 1854 Ceded Territory [1]. Moose (mooz; *Alces alces*) and white-tailed deer (waawaashkeshi; *Odocoileus virginianus*) are primary subsistence species of Anishinaabe people. Gray wolves (ma’iingan; *Canis lupus*) are culturally and environmentally important to their seventh-generation planning philosophy of environmental stewardship [2]. The Grand Portage Band conducts predator–prey research to improve their understanding of ecosystem health, which set the context for this study.

### Migratory coupling

A primary assumption of predator–prey movement modeling is that predators have good spatial memories, otherwise prey could remain in high quality patches indefinitely because predators would not concentrate space use in these areas [3, 4]. Prey are therefore less likely to experience predation if their movements among resource patches are unpredictable [3]. However, seasonal migrations can reduce variation in inter-individual movements resulting in predictable population-level responses [5, 6]. When seasonal second-order habitat selection (i.e., home range selection [7]) by predators matches these predictable prey movements, migratory coupling occurs [8]. Predator home range shifts considered migratory coupling can vary from fine-scale shifts (e.g., seasonal use of migratory bottlenecks or feeding grounds) to complete migration by predators to follow prey [8]. These shifts generally lead to increased predation risk among migrating prey [8].

Evolutionarily, prey should not migrate if the costs outweigh the benefits [9]. Two primary benefits of migration are increased forage opportunities and decreased seasonal predation risk [9, 10]. Migrating to match available forage is common among mammals [9]; African elephants (*Loxodonta Africana*) [11], red deer (*Cervus elaphus*) [12], and bats (order Chiroptera) [13] demonstrate migratory behavior for foraging. Though more difficult to identify, migrating to reduce seasonal predation risk also occurs among mammals (e.g., baleen whales [parvorder Mysticeti] [14], and bighorn sheep [*Ovis canadensis*] [15]) [9].

Despite the benefits, migration often increases predation risk for prey. Predation risk of migrating wildebeest (*Connochaetes taurinus*), for example, increased during migration due to foregoing predator avoidance in favor of high quality forage [16], and pronghorn (*Antilocapra americana*) experienced increased predation risk from mountain lions (*Puma concolor*) when migrating through typically unused and narrow forest corridors [17]. Prey communities must respond to tradeoffs between predation risk and improved forage access to maintain the benefits of migration [9].

Gray wolves are obligate carnivores [18, 19] whose space use can alter predation risk and increase mortality of migrating prey. Caribou (*Rangifer tarandus*) predation by wolves in Finland increased during their migrations [20]. Migratory elk (*Cervus canadensis*) in Yellowstone National Park, USA decreased predation risk by migrating but were about 1.7 times more likely to experience predation during migration, and 63% of migratory elk deaths occurred during or immediately before or after migration [21, 22]. Non-territorial wolves in tundra ecosystems exhibited migratory coupling by following caribou populations during their seasonal migrations [23, 24]. Comparatively, wolves in boreal ecosystems maintain territories year-round [25] but may alter second-order habitat selection in response to seasonal prey space use [26].

Despite predation risk generally increasing during migration in the presence of territorial predators [20, 27], the spatial response of territorial predators to prey migration remains less understood [8]. Ungulates (i.e., moose and white-tailed deer) are primary gray wolf prey [18] and invaluable subsistence species for Indigenous peoples. The Grand Portage Band of Lake Superior Chippewa has been conducting predator–prey research toward effective stewardship of subsistence resources [28–30]. We furthered this research by investigating the response of a territorial predator, gray wolves, to migration and seasonally shifting spatial distributions of prey. We hypothesized wolves would shift their ranges to increase use of ungulate migration corridors during migrations as predicted by migratory coupling [8]. We predicted seasonal wolf movements would overlap with primary available ungulate prey.

## Methods

### Study area

**Fig. 1:**
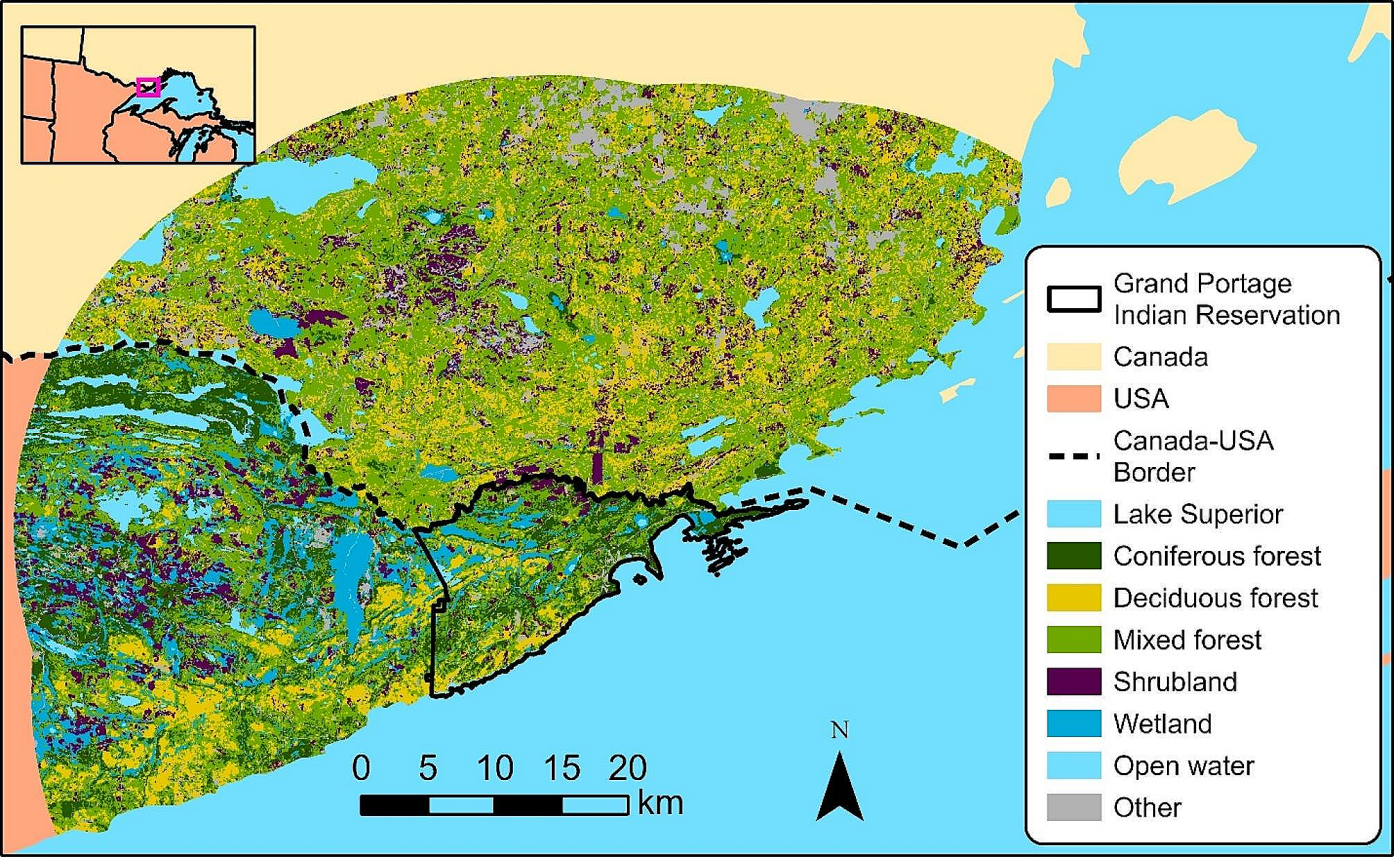
Study area on and near the Grand Portage Indian Reservation, Minnesota, USA and adjacent portions of Ontario, Canada. Land cover data is from the Commission for Environmental Cooperation 30-m land cover map of North America [32]

The Grand Portage Indian Reservation (196 km^2^) is in northeastern Minnesota, USA (47.9614° N, 89.7594° W). The reservation borders Lake Superior to the southeast, Ontario, Canada to the north, and U.S. federal, state, and private properties to the west. Our approximately 1,200 km^2^ study area included the reservation and mainland areas within 30 km (Fig. 1). Elevations are 183–674 m above sea level with broad valleys between steep ridges [31]. The area contains 11% coniferous forest, 17% deciduous forest, 44% mixed forest, 9% shrubland, 7% wetland, and 5% open water [32]. Temperatures within the study area (Cook County, Minnesota, USA) during 2009–2019 ranged from mean daily minima of -17.8 ± 3.5 C° (mean ± SD) in January to mean daily maxima of 23.3 ± 1.7 C° in July; annual precipitation included 83.8 ± 11.7 cm of rainfall and 150.2 ± 80.8 cm of snowfall [33].

The primary prey of gray wolves in the western Great Lakes region are white-tailed deer, moose, and American beaver (*Castor canadensis*) [34, 35]. About 80–95% of the region’s deer migrate and exhibit winter and summer range fidelity [36, 37]. Moose in the region are semi-nomadic with about 20% migrating between summer and winter ranges while the remainder maintain a single year-round home range or shift among multiple ranges without clear patterns [38]. Wolves could be legally harvested in Ontario during the study period [39], but legal harvest in Minnesota occurred only during 2012–2014 [40]. Deer and moose could also be legally harvested, but after 2013, only Indigenous band members could harvest moose in Minnesota [41, 42]. Wolves were not harvested by Grand Portage Band members when legally permitted.

### Analytical approach

We tested our hypothesis that gray wolves would shift their ranges to increase use of migration corridors during migration and our prediction that wolves would concentrate movements on seasonally available prey in three stages. First, we determined home ranges, movement strategies, movement timing, seasonal population-level occurrence distributions, and locations of migration corridors using Brownian bridge movement models (BBMMs) in Migration Mapper (v3.0) [43]. Potential movement strategies included resident (single year-round home range), migratory (seasonally shifting between 2 and 3 home ranges), nomadic (shifting among ≥ 4 home ranges), or unknown (movement strategy could not be assessed). We recorded movement timing as the dates during which animals moved between seasonal home ranges. Population-level occurrence distributions are estimates of where a population is likely to be in a given time period based on individual-level occurrence distributions, but they do not represent home ranges [44]. Second, we reassessed whether wolves exhibited home range shifts and, if so, the timing and duration of those home range shifts using mechanistic range shift analysis (MRSA) to corroborate our Migration Mapper results [45]. Third, we calculated individual-level utilization distributions (UDs) for wolves and overlap of these UDs with population-level occurrence distributions of prey and their migration corridors and tested for seasonal differences using multivariate linear models.

We used GPS location data from gray wolves, moose (each monitored October 2012–December 2021), and white-tailed deer (monitored March 2016–December 2021) collared during research conducted by the Grand Portage Band of Lake Superior Chippewa Department of Biology and Environment [28]. Wolves were captured using foothold traps [46], moose using aerial darting [47], and deer using clover traps [48]. Capture and handling protocols were approved by the Grand Portage Band of Lake Superior Chippewa Tribal Council, Michigan State University institutional animal care and use committee (IACUC) (PROTO202200266), and State University of New York College of Environmental Science and Forestry IACUC (210702). Collar relocation data was obtained via satellite or, upon retrieval, downloaded from the collar. Minimum relocation intervals of wolf, deer, and moose collars were 3.25–4.5 h (depending on estimated collar longevity), 4 h, and 0.25–4 h (depending on time of year), respectively. We resampled moose locations to 4-h intervals using the R package *padr* [49] to make them similar to wolf and deer relocation intervals. We excluded from analyses individuals that dispersed from the study area.

### Migration Mapper

Migration Mapper is a software application that allows users to analyze GPS location data using six modules in a web browser interface while underlying calculations occur in the R statistical platform [43, 50]. We used Migration Mapper to determine home ranges, movement strategies, and timing as well as population-level occurrence distributions and migration corridors for gray wolves, moose, and white-tailed deer. Below, we report the decisions we applied while using this software; for a complete guide to the software see Merkle et al. [43] and their associated user guide and videos. We used default settings unless otherwise stated.

In Module 1, we censored anomalous locations from our dataset. Migration Mapper identified locations as potentially anomalous if their movement distances were < 50 m in 48 h because Migration Mapper considered these locations to have been recorded after the animal had died. However, we retained these locations because we censored locations occurring after animal mortality before data were imported to Migration Mapper. Migration Mapper also identified locations as potentially anomalous if their movement speeds were > 10.8 km/h between consecutive 4-h relocations; we censored these locations as we considered these movement speeds implausible. We manually inspected remaining locations using first passage time (i.e., the time an animal requires to cross a circle of defined radius, which describes the relative use of an area [51]) and relative turning angle, and we censored locations exhibiting small first passage time values (≤ 1, indicating rare usage [51]) and tight turning angles (179–181°, indicating direct routes to and from the location) because these locations were likely anomalous.

In Module 2, we visually identified home ranges, migratory movements between them, and migration timing using maps of locations and temporal graphs of net squared displacement and movement speed. We set the beginning of the monitoring period to 13 February (the earliest capture date for white-tailed deer); if an individual was monitored for more than one year, its movement status (i.e., migratory, resident, nomadic, or unknown) was aggregated across all monitored years. When individuals moved between winter and summer ranges multiple times, we considered individuals to have migrated during the movement period associated with the longest stay in either range. We assessed migratory status following examples from Migration Mapper [43]. Specifically, we considered stopover events (i.e., animals stopping in a single concentrated area along their migration route for < 30 days) part of the migration event, and we considered individuals nomadic, but not migratory, if they moved among ≥ 4 distinct home ranges at irregular intervals. If an individual was not monitored long enough (the duration of one migration season for individuals with typical movements) to visually assess its movement status due to collar failure or mortality, we considered its movement status unknown. If an individual was migratory, we recorded the displacement (i.e., Euclidian distance) between the arithmetic centroids of its winter and summer home ranges; if an individual did not exhibit range fidelity, we averaged its displacement measures.

In Module 3, we categorized location data into seasons using animal movements. We considered spring and fall migration to occur from the first quartile date migratory white-tailed deer departed for their new seasonal range to the third quartile date migratory deer arrived in their new seasonal range (Table 1). We defined summer and winter as the periods between spring and fall migration. We used the same seasons (winter, spring migration, summer, and fall migration) for all species to compare population-level occurrence distributions across species and time periods. We used deer migrations to define seasons because gray wolves and moose did not exhibit seasonally-defined movements (see results).

In Module 4, we used BBMMs from the R package *BBMM* [52] to generate individual-level occurrence distributions for compilation into population-level occurrence distributions for each season in Module 5. We allowed Migration Mapper to calculate movement variance incorporating individual movement behaviors instead of manually selecting a movement variance value. We specified a 50-m resolution for distributions estimated by Migration Mapper (default = 500-m resolution) to enhance occurrence distribution resolutions, and we increased the maximum lag time (i.e., the time interval between relocations) to 9 h to allow for a single missed relocation.

In Module 5, we merged individual-level occurrence distributions to form seasonal population-level occurrence distributions. We compiled a model for each season for gray wolves, moose, and three subsets of white-tailed deer: migratory individuals only, resident individuals only, and all individuals combined (the composite population). This hierarchical process produced 20 population-level seasonal occurrence distributions representing the four seasons and five populations. We produced population-level distributions by (1) calculating mean distributions of individuals during the specified season by merging their individual-level distributions from Module 4, (2) calculating mean season- and year-specific population distributions by merging mean distributions of all individuals monitored in a specified year, and (3) calculating final population-level occurrence distributions across the duration of the study by merging mean season- and year-specific distributions. We generated one additional model to represent the deer migration corridor by repeating the above three steps with an added step (2.5) in which we merged spring and fall season- and year-specific population distributions. We merged the spring and fall migration corridors as they were highly similar (93.5% overlap; calculated following Cardillo and Warren [53]). Unlike seasonal population-level distributions, we treated the migration corridor distribution as a single distribution that did not change seasonally. Finally, we exported the 21 resultant 95% population-level occurrence distributions from Migration Mapper as shapefiles. We used Module 6 for data visualization throughout the process.

**Table 1:** Timing of annual migrations by white-tailed deer (*Odocoileus virginianus*) on and near the Grand Portage Indian Reservation, Minnesota, USA, 2016–2021

Migration event	5% start migrating	25% start migrating	Average migration start	Average migration finish	75% finish migrating	95% finish migrating
Spring	2 April	4 April	19 April	28 April	2 May	2 June
Fall	11 August	10 October	28 October	8 November	28 November	13 January

### Mechanistic range shift analysis (MRSA)

We tested if gray wolves shifted their home ranges using MRSA in the *marcher* package [45, 54] in R (v4.2.1) [50] to corroborate results from Migration Mapper. The utility of MRSA is the statistical validation of the occurrence of range shift behaviors. We visually searched the movements of each wolf for temporal differences in latitude and longitude and applied a 3-cluster means process including three of the four seasons observed to identify potential home range shifts [45, 54]. We then fit a migratory white noise range shift model using maximum likelihood and tested for statistical significance (α < 0.05) [45, 54]. If a model identified a home range shift, we recorded the estimates of range shift timing and duration with 95% confidence intervals (CI).

### Overlap calculations

We assessed whether gray wolves switched prey seasonally by calculating spatial overlap of wolves with prey using methods similar to Michelot et al. [24]. We calculated gray wolf utilization distributions using kernel BBMMs [55]. We excluded wolves if < 5% of recorded locations were within the 95% white-tailed deer migration corridor occurrence distribution outer boundary due to dispersal or home range location. We subset remaining wolf locations into the four seasons defined using Migration Mapper (winter, spring migration, summer, and fall migration). We removed wolf-seasons with < 50 locations, which represented one week of monitoring and the approximate minimum time required by wolves to traverse their home ranges several times as determined visually using variograms in the R package *ctmm* [56, 57]. We calculated the outer boundary of 95% kernel BBMM UDs for each wolf-season in the R package *adehabitatHR* [24, 58]. We calculated the proportion of the area within those boundaries overlapping the corresponding population-level seasonal prey occurrence distributions and the deer migration corridor (hereafter, proportional overlap) in the R package *sf* [24, 59].

We used multivariate linear models [60] to assess whether proportional overlap of gray wolves with moose and white-tailed deer distributions changed across seasons. We used five models to assess each prey distribution individually (Model 1 = moose, Model 2 = deer migration corridor, Model 3 = composite deer population, Model 4 = migratory deer only, Model 5 = resident deer only). Each model fit proportional overlap of wolves with the specified prey distribution against season with sex and pack affiliation as nuisance covariates. We logit-transformed proportional overlap in all models because the response was a [0, 1] bounded proportion; we considered all proportions > 0.975 and < 0.025 to be 0.975 and 0.025, respectively, because logit(1) and logit(0) are infinite [61]. Sex was determined for each wolf at time of capture. Wolf pack affiliation included four possible categories; the first three were northeast (NE), northwest (NW), and southwest (SW) as determined by the portion of the migration corridor in which the pack resided (Fig. S1). The final affiliation was floating (FL), which was assigned to wolves with year-round home ranges > 561 km^2^ as their movements could not be classified as resident [62]. Finally, we used Mann-Whitney tests to identify differences in proportional overlap with wolves seasonally between prey populations [63].

## Results

### Movement Status

We obtained location data from 45 gray wolves (median = 623 locations per individual; range = 7–2,721). Of these, we assessed seasonal movement status of 35 wolves (sex: females = 19, males = 16; pack affiliation: NE = 7, NW = 9, SW = 10, FL = 9). We did not assess the movement status of 10 wolves: 3 wolves that recorded too few locations and 7 wolves because < 5% of their total recorded locations were within the deer migration corridor of which 6 dispersed from the study area. Most wolves were captured during July–October; consequently, the periods of greatest wolf monitoring were summer, fall, and winter with relatively few collars active during spring migration (Fig. S2). We monitored 3 wolves for 2 years, including one wolf that was captured and collared twice. No wolves exhibited range shifts or were migratory; instead, all wolves maintained a single home range during their respective monitoring periods.

**Fig. 2:**
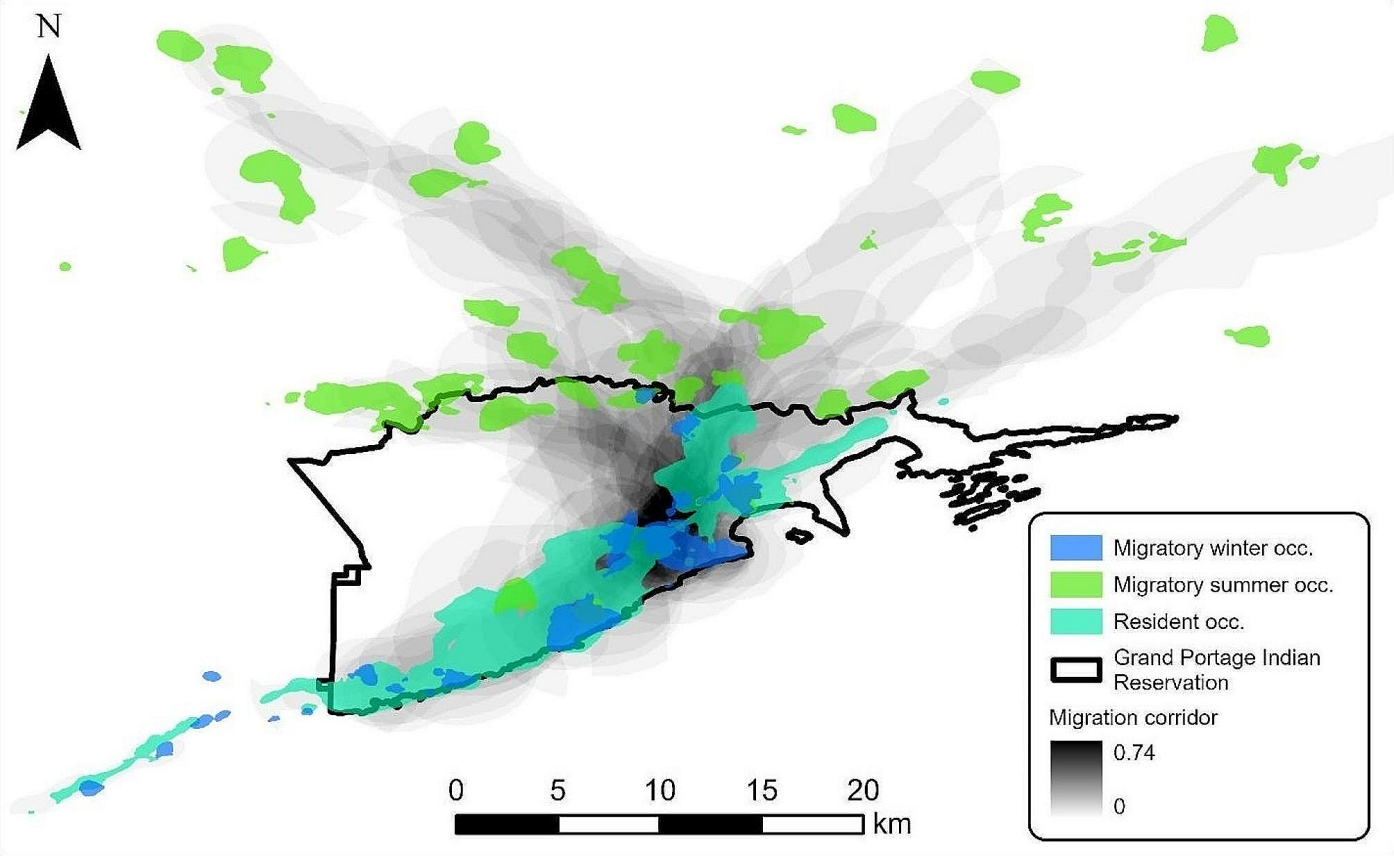
White-tailed deer (*Odocoileus virginianus*) migration corridor on and near the Grand Portage Indian Reservation, Minnesota, USA, 2016–2021. Blue and green polygons represent 95% occurrence distributions of the migratory deer population during winter and summer, respectively; gray-scale overlapping polygons represent the proportion of migratory deer using a given area during their spring and fall migration movements between winter and summer home ranges; teal polygons represent 95% year-round occurrence distributions of resident deer

We obtained data from 135 moose (median = 2,541 locations per individual; range = 6–16,042) and 72 white-tailed deer (median = 1,363; range = 6–8,497). We monitored 85 moose for 2–10 years, and 35 deer for 2–5 years. We were unable to assess the movement status of 29 moose and 9 deer that dispersed from the study area or were not monitored long enough to assess movement status due to mortality or collar failure. In total, we assessed movement status of 106 moose and 63 deer. Among moose, 58 (54.7%) were nomadic, 11 (10.4%) migrated, and 37 (34.9%) maintained a single home range. Median displacement between migratory moose winter and summer home ranges was 5.0 km (range = 1.5–20.0 km), but we did not identify a moose migration corridor. Among deer, 42 (66.7%) migrated and 21 (33.3%) maintained a single home range. We identified 62 spring and 46 fall deer migration events from 42 individuals including 15 individuals monitored for 2–5 years exhibiting migration each year. Spring migration by deer primarily occurred synchronously in April, and fall migration primarily occurred asynchronously during October–November (Table 1; Fig. S3; Fig. S4). Migratory deer followed a single migration corridor between their winter and summer ranges (Fig. 2). Median displacement between migratory deer winter and summer home ranges was 16.8 km (range = 3.5–33.0 km).

### Seasonal overlap

We calculated population-level occurrence distributions for moose-seasons and white-tailed deer-seasons as well as separate distributions for migratory and resident deer during winter (214 moose, 45 deer [32 migratory, 13 resident]), spring migration (268 moose, 104 deer [69 migratory, 35 resident]), summer (256 moose, 96 deer [67 migratory, 29 resident]), and fall migration (213 moose, 73 deer [55 migratory, 18 resident]). We calculated individual-season models for gray wolves during winter (*n* = 33), spring migration (*n* = 11), summer (*n* = 25), and fall migration (*n* = 32).

**Fig. 3:**
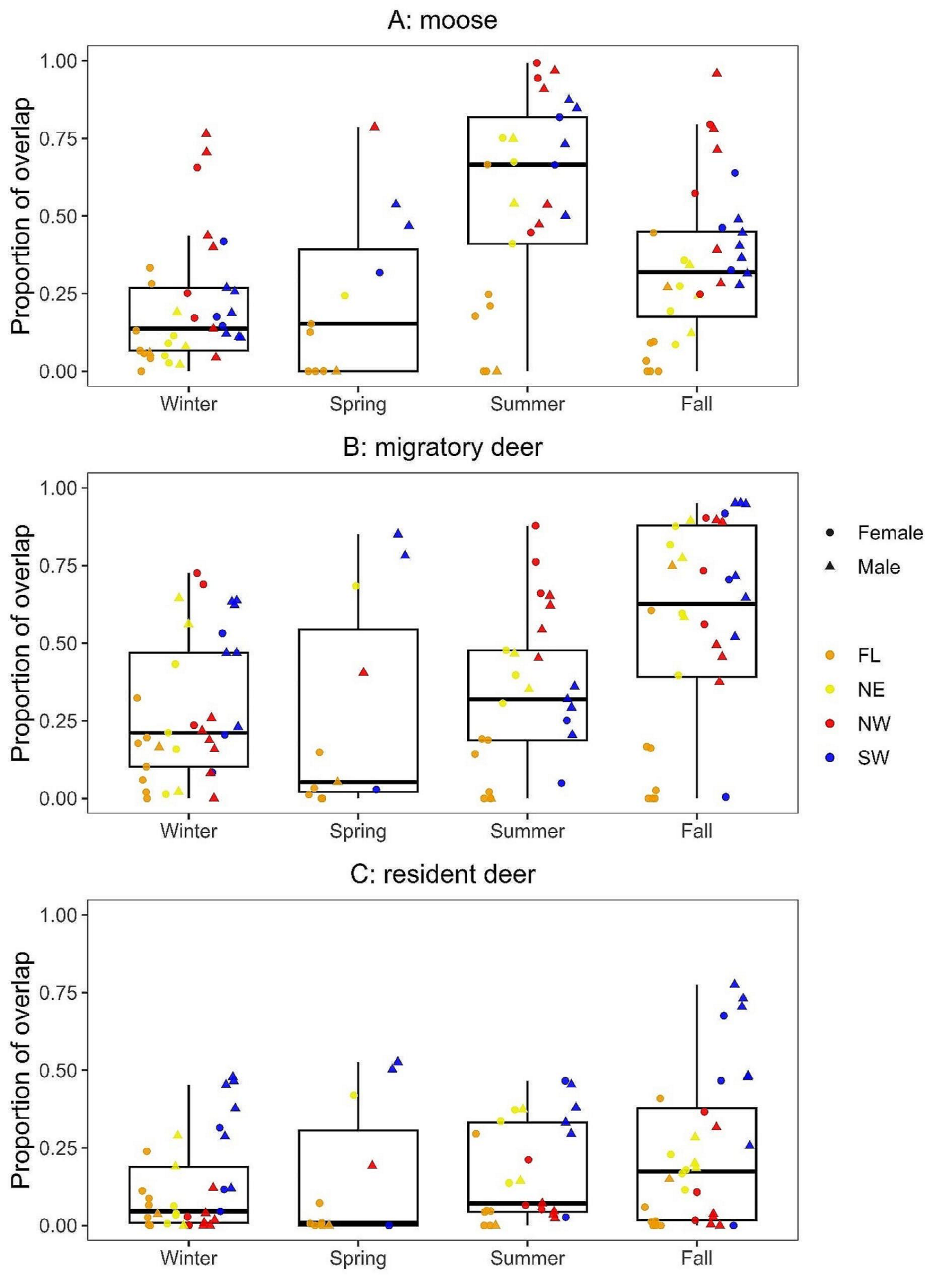
Seasonal proportional overlap of gray wolves (*Canis lupus*) with prey on and near the Grand Portage Indian Reservation, Minnesota, USA, 2012–2021. Proportion of overlap was calculated using 95% wolf UDs and 95% occurrence distributions of (**A**) moose (*Alces alces*), (**B**) migratory white-tailed deer (*Odocoileus virginianus*), and (**C**) resident deer. Points represent proportion of overlap for each wolf monitored during winter, spring migration (spring), summer, and fall migration (fall). Individual wolf characteristics are indicated where circles represent females, triangles represent males, and colors indicate pack affiliations (FL = floating, NE = northeast, NW = northwest, and SW = southwest).

Proportional overlap of wolf UDs with moose distributions differed among seasons and was greatest during summer (Fig. 3A; Table 2). Overlap of gray wolf UDs and the white-tailed deer migration corridor was lower during spring migration (Fig. S5). Overlap of wolf UDs with combined migratory and resident deer distributions changed seasonally and was greatest during fall migration. Overlap of wolf UDs with these composite deer distributions was greater than that of wolves with moose during fall migration (Table 3). Proportional overlap of wolf UDs with only migratory deer occurrence distributions changed seasonally and was greatest during fall migration (Fig. 3B). Overlap of wolves with only resident deer distributions changed seasonally and was marginally greater during fall (Fig. 3C). Overlap of wolves with only migratory deer was greater than overlap with only resident deer during summer, fall migration, and winter. Pack affiliation influenced overlap such that floating wolves overlapped all prey distributions less than resident packs. Wolves in the NW pack also overlapped resident deer less than other packs. Wolf sex did not influence spatial overlap.

**Table 2:** Multivariate linear model coefficients assessing overlap of gray wolves (*Canis lupus*) with prey on and near the Grand Portage Indian Reservation, Minnesota, USA, 2012–2021. Comparisons of logit-transformed proportional overlap of individual-level wolf 95% UDs with population-level 95% occurrence distributions of moose (*Alces alces*), the white-tailed deer (*Odocoileus virginianus*) migration corridor, the composite deer population, migratory deer only, or resident deer only are presented

	Model 1:				Model 2:				Model 3:				Model 4:				Model 5:		
	moose				deer migration corridor				all deer				migratory deer				resident deer		
Covariates	β	SE	*p*		β	SE	*p*		β	SE	*p*		β	SE	*p*		β	SE	*p*
Season: spring^a^	0.66	0.41	0.12		-1.43	0.61	0.02		0.07	0.54	0.90		0.24	0.51	0.64		0.27	0.38	0.48
Season: summer^a^	2.05	0.31	< 0.01		-0.44	0.46	0.34		0.61	0.40	0.13		0.40	0.38	0.30		0.51	0.28	0.08
Season: fall^a^	0.86	0.29	< 0.01		0.11	0.43	0.80		1.50	0.38	< 0.01		1.53	0.36	< 0.01		0.71	0.27	< 0.01
Sex: male^b^	-0.07	0.26	0.78		0.75	0.39	0.06		0.43	0.34	0.21		0.41	0.32	0.21		0.46	0.24	0.06
Pack: NE^c^	1.20	0.35	< 0.01		3.63	0.52	< 0.01		2.12	0.46	< 0.01		2.11	0.43	< 0.01		1.14	0.32	< 0.01
Pack: NW^c^	2.98	0.35	< 0.01		2.71	0.52	< 0.01		2.18	0.45	< 0.01		2.27	0.43	< 0.01		-0.13	0.32	0.68
Pack: SW^c^	2.16	0.34	< 0.01		3.11	0.51	< 0.01		2.46	0.45	< 0.01		2.16	0.42	< 0.01		2.01	0.32	< 0.01

^a^Winter was used as the reference category for season; ^b^female was the reference category for sex; ^c^FL was the reference category for pack affiliation

**Table 3:** Mann-Whitney tests for differences in proportional overlap of gray wolves (*Canis lupus*) with prey on and near the Grand Portage Indian Reservation, Minnesota, USA, 2012–2021. Population comparisons represent proportional overlap of individual-level wolf 95% UDs (n) with population-level 95% occurrence distributions of moose- (*Alces alces*) seasons (N) and resident, migratory, and composite white-tailed deer- (*Odocoileus virginianus*) seasons (N). A significant test (*p* < 0.05) indicates proportional overlap of wolves with specified populations differed during the specified season; the population with greater overlap is designated in the final column

n (wolves)	Population 1 (N)	Population 2 (N)	Season	W	*p*-value	Greater overlap
33	Composite deer (45)	Moose (214)	Winter	673	0.10	N/A
11	Composite deer (104)	Moose (268)	Spring	64	0.84	N/A
25	Composite deer (96)	Moose (256)	Summer	217	0.06	N/A
32	Composite deer (73)	Moose (213)	Fall	743	< 0.01	Composite deer
33	Resident deer (13)	Migratory deer (32)	Winter	794	< 0.01	Migratory deer
11	Resident deer (35)	Migratory deer (69)	Spring	76	0.32	N/A
25	Resident deer (29)	Migratory deer (67)	Summer	431	0.02	Migratory deer
32	Resident deer (18)	Migratory deer (55)	Fall	795	< 0.01	Migratory deer

## Discussion

Gray wolves in our study did not exhibit home range shifts or migratory coupling, but did have greater spatial overlap with white-tailed deer during fall migration partially supporting our hypothesis. Though migratory coupling has occurred among non-territorial migratory wolves pursuing migratory caribou [24, 64]. Non-migratory wolves, like those in our study, are territorial and defend their home ranges year-round [25]. Spatial constraints due to intraspecific territoriality likely explain why wolves in our study did not shift their ranges seasonally. Migratory coupling has only been reported among non-territorial (e.g., grizzly bear [*Ursus arctos*] [65, 66]), semi-territorial (e.g., cheetah [*Acinonyx jubatus*] [67, 68]), or seasonally territorial (e.g., red knot [*Calidris canutus*] [69, 70]) predators [8]. This pattern indicates territorial predators may not exhibit migratory coupling as proposed by Furey et al. [8]. An exception may occur among territorial wolves whose ranges were seasonally limited by elevation-mediated snow depths [26], but this requires further examination.

Contrasting our hypothesis, gray wolf distributions overlapped the migration corridor similarly year-round, excepting spring migration. Migratory corridors typically follow least-cost paths that facilitate animal movement [71], and wolves select for least-cost paths to increase prey encounters [72, 73]. Though we did not assess landscape resistance, wolves could be using the corridor year-round to optimize foraging. Alternatively, resource dispersion hypothesis posits predators should defend the minimum amount of territory necessary to support themselves when prey are least available, which may include maintaining access to migration corridors even when prey are not migrating [74]. Wolves, cheetahs, and African lions (*Panthera leo*) exhibited such behavior despite territorial limitations [67, 75]. Wolves may therefore maintain access to the migration corridor year-round to access increased prey availability during migrations [74] or to facilitate improved mobility in all seasons [72, 76].

Though we did not observe home range shifts or migratory coupling, our prediction that gray wolves would adapt to seasonal prey availability was supported as wolves altered their within-range spatial distributions in concert with seasonal prey distributions. This result contrasts assertions that wolves alter prey distributions rather than responding to them [28]. A possible explanation is that both patterns occur simultaneously; we used prey to describe wolf movement whereas Oliveira-Santos et al. [28] used wolves to describe prey movement. We observed greater overlap of wolves with migratory white-tailed deer during fall migration as well as with moose during summer. This outcome suggests a spatial response to memories of predation success and biased movements towards available prey in support of prey switching under alternative prey hypothesis [3, 77, 78]. Seasonal prey switching in response to relative prey availability is common among predators [79, 80]. In our study area, migratory deer were present in winter while moose were present year-round. Further, fall migration by deer is protracted and asynchronous while spring migration is brief and synchronous. Analogous to prey switching, wolves should concentrate their spatial distributions on the more functionally available deer in fall and winter then switch to the best alternatives in summer when deer are less abundant [81]. This pattern is supported by the greater proportions of deer in wolves’ winter diets and of beaver and moose calves in summer diets [35, 82] as well as increased deer mortality during fall migration due to wolf predation [30].

Migratory white-tailed deer in our study experienced the greatest overlap with gray wolves during fall migration. Prey migration is risky due to decreased vigilance and lessened familiarity with areas traversed [16, 21, 83]; however, migration to reduce predation risk or seek better forage is common [9]. Though overlap may not equate to risk [84, 85], deer in our study are likely most vulnerable during fall migration because their asynchronous and predictable movements could facilitate higher predation success [3]. Deer may also exhibit reduced predator avoidance behaviors (e.g., diurnal activity) during the rut, which coincides with fall migration [36, 73], though male ungulates may exhibit greater vigilance during this period [86]. Comparatively, spring deer migration was synchronous, which may have limited wolves’ ability to respond to spring migration movements [87]. Wolves also reduce movements during denning and parturition, which occurred during spring deer migration [88, 89]; however, lower proportional overlap of wolves with the migration corridor during this period in our study is likely an artefact of our low sample size and the predominance of floating wolves therein. We suggest increased overlap of wolves with migratory deer during fall is a consequence of increased predation success due to greater deer vulnerability and increased availability of deer carcass remains from hunter harvest. Supporting our conclusions, deer and caribou experienced greater mortality during fall migration than during spring migration [20, 30], and wolves used anthropogenic sources of carrion [90]. Our models, however, may not have identified complete patterns as wolves could have pursued unmonitored deer whose fall migrations were not examined or corridor distributions may have been too coarse due to the 4–8-h relocations used in our analyses [43].

Compared to fall migration, white-tailed deer exhibited lower overlap with gray wolves during winter suggesting potential predator avoidance when they are in poorest condition [91, 92]. Migratory deer also experienced greater overlap with wolves year-round than resident deer. Migratory elk increased predation risk to obtain greater summer forage while resident elk exhibited the opposite [93, 94]. Migratory deer in our study may do the same and exhibit increased predator avoidance in winter and forage seeking in summer [9, 73]. Resident deer in our study remained closer to Lake Superior shoreline habitats that receive less snowfall and have greater human activity associated with increased housing density and traffic from the region’s main thoroughfare [95, 96]. These factors may reduce risk through increased deer mobility and human shield effect [97, 98] and may explain why a lower percentage of deer in our study were migratory than in nearby inland populations [36, 37].

We observed an increase in gray wolf and moose overlap during summer despite the relative year-round range stability of these species. There are several possible explanations for this response. First, many white-tailed deer, which are wolves’ primary prey [35, 99], are absent from the core of our study area during summer. Second, moose calves are spatially concentrated in predictable landscape-level patterns during summer, more vulnerable to predation, and have high mortality rates due largely to wolf predation [29, 82]. Third, American beaver account for up to one third of summer wolf biomass consumption [35, 99], and resource selection by wolves suggests selection for beaver habitat [89, 100]. Finally, wolves select for flatter slopes in mid- to late summer when pups are immature and less mobile [26, 89]. This combination of decreased deer availability, high moose calf vulnerability, increased beaver availability, and limited wolf pup mobility likely explains the greater spatial overlap of wolves and moose in summer.

### Management implications

Moose populations are declining throughout their southern range including the 1854 Ceded Territory [101, 102]. The Grand Portage Band desires to increase moose abundance and conducts management to limit population declines. For example, implementing spring black bear (*Ursus americanus*) harvests appears to have improved moose recruitment (Grand Portage Band of Lake Superior Chippewa, *unpublished data*). Our results and previous work indicate moose likely experience greater gray wolf predation pressure during summer when calves are more vulnerable [82, 103]. If management goals include further increases in moose calf recruitment, management actions to reduce wolf predation of calves could be implemented [104, 105], though Indigenous constituents’ opinions should also be considered [106].

## Conclusions

Our work is among the first to use season-specific population-level occurrence distributions for analysis of predator–prey interactions [24, 44]. Gray wolves seasonally altered their within-range spatial distributions supporting prey switching. We also demonstrate spatial plasticity of predators in response to spatially dynamic prey, which is well-studied among non-territorial predator populations [8] but warrants further consideration among territorial and semi-territorial species [67, 75]. Wolves in our study did not, however, exhibit home range shifts or migratory coupling in response to white-tailed deer migration. Because wolves in the western Great Lakes region are territorial with little available space between packs [25, 107], even subtle range shifts between seasons may be inhibited. We suggest territorial predator populations can exhibit within-territory shifts in spatial distributions but not migratory coupling as originally postulated by Furey et al. [8], though an exception may occur among territorial predators whose ranges are seasonally limited by weather conditions (e.g., snow depth) [26].

### Electronic supplementary material

Below is the link to the electronic supplementary material.

Supplementary Material 1

Supplementary Material 2

Supplementary Material 3

## Data Availability

Data used for this project has not been made publicly available to protect tribal data sovereignty. To request access, please contact the Grand Portage Band of Lake Superior Chippewa.

## Abbreviations

BBMM: Browning bridge movement model
CI: Confidence intervals
FL: Floating
IACUC: Institutional animal care and use committee
MRSA: Mechanistic range shift analysis
NE: Northeast
NW: Northwest
SW: Southwest
UD: Utilization distribution
