# Supplementary figures and images for: Spatial overlap of gray wolves and ungulate prey changes seasonally corresponding to prey migration
Source: Mov Ecol. 2024 Apr 26;12:33. doi: 10.1186/s40462-024-00466-w (PMC11046751; doi:10.1186/s40462-024-00466-w)

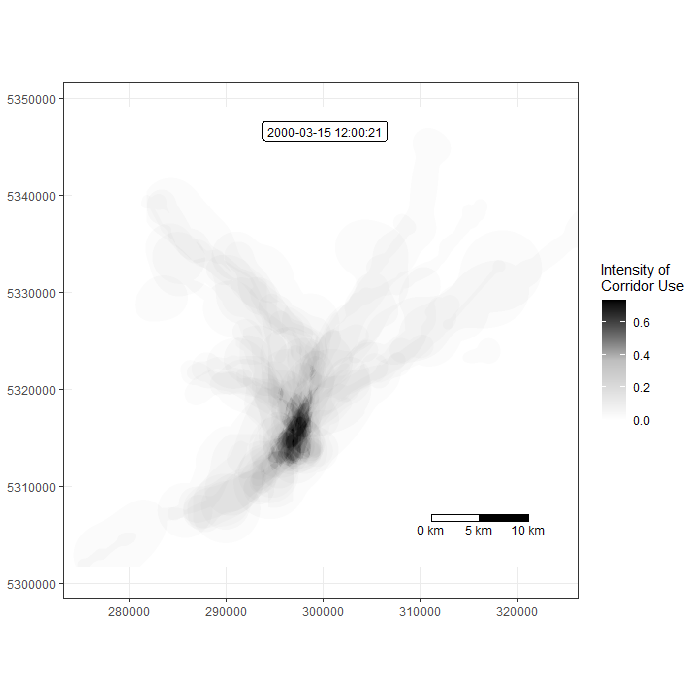

Supplement: Supplementary file 1 — Supplementary Material 1 [file 40462_2024_466_MOESM1_ESM.gif]

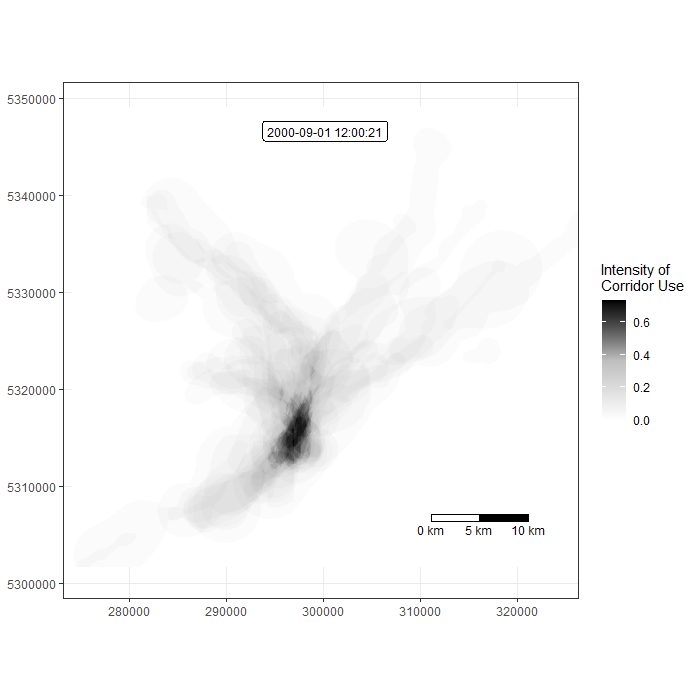

Supplement: Supplementary file 2 — Supplementary Material 2 [file 40462_2024_466_MOESM2_ESM.gif]
