# Supplementary material for: Spatial overlap of gray wolves and ungulate prey changes seasonally corresponding to prey migration
Source: Mov Ecol. 2024 Apr 26;12:33. doi: 10.1186/s40462-024-00466-w (PMC11046751; doi:10.1186/s40462-024-00466-w)

**Fig. S1** Gray wolf (*Canis lupus*) home ranges on and near the Grand Portage Indian Reservation, Minnesota, USA, 2013–2021. Wolf identifiers include sex (M = male, F = female) and 7-digit individual ID. Polygon color indicates pack affiliation (yellow = northeast, red = northwest, blue = southwest). Floating wolves with no pack affiliation are excluded. The year indicates the year during which most of the wolves’ locations were recorded; no pack-affiliated wolves were predominantly monitored during 2017. Basemaps include World Hillshade and World Topographic Map from ArcGIS Pro (v3.0.3, Esri, Redlands, California, USA).

**
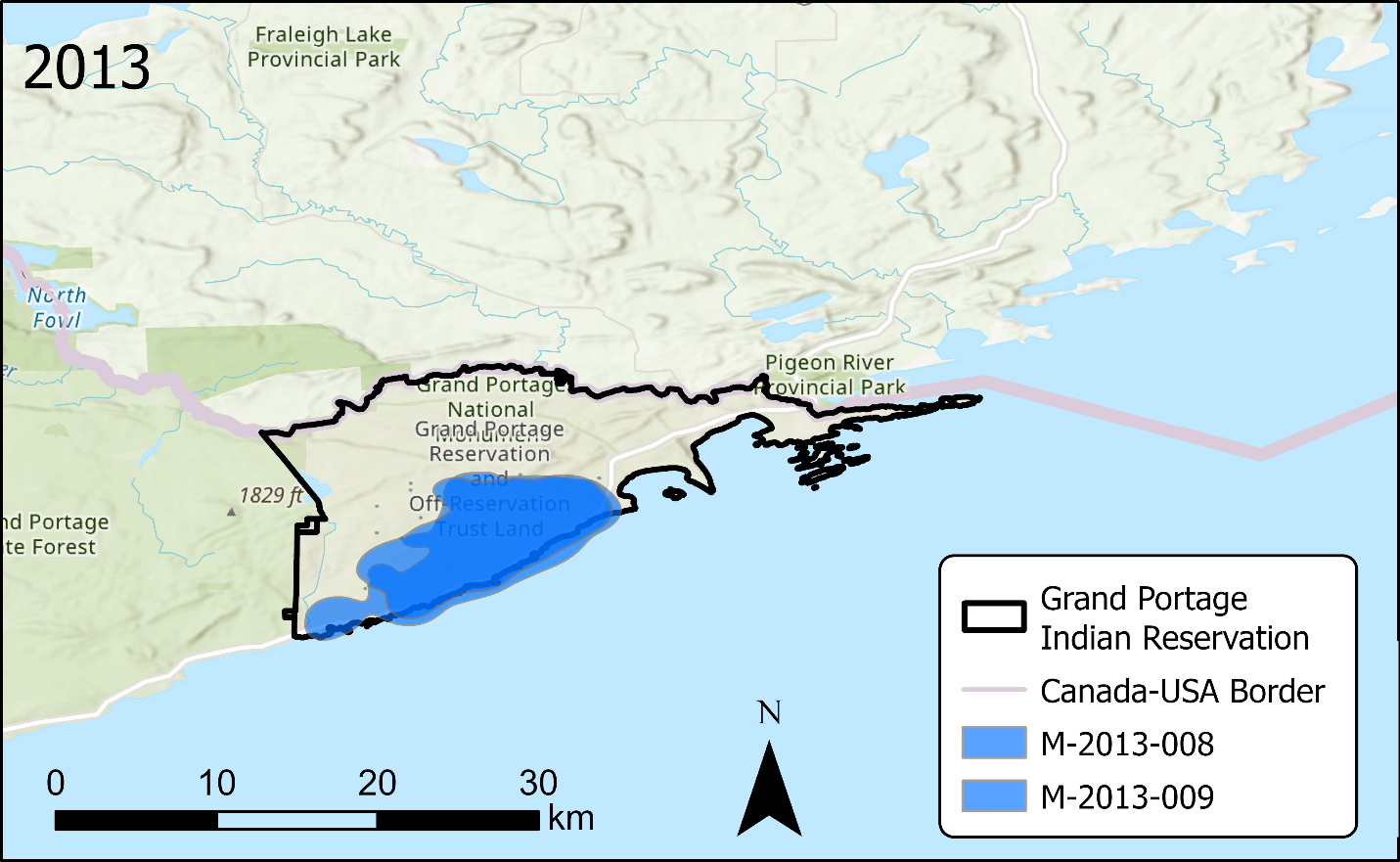
**

**Fig. S1** Continued

**
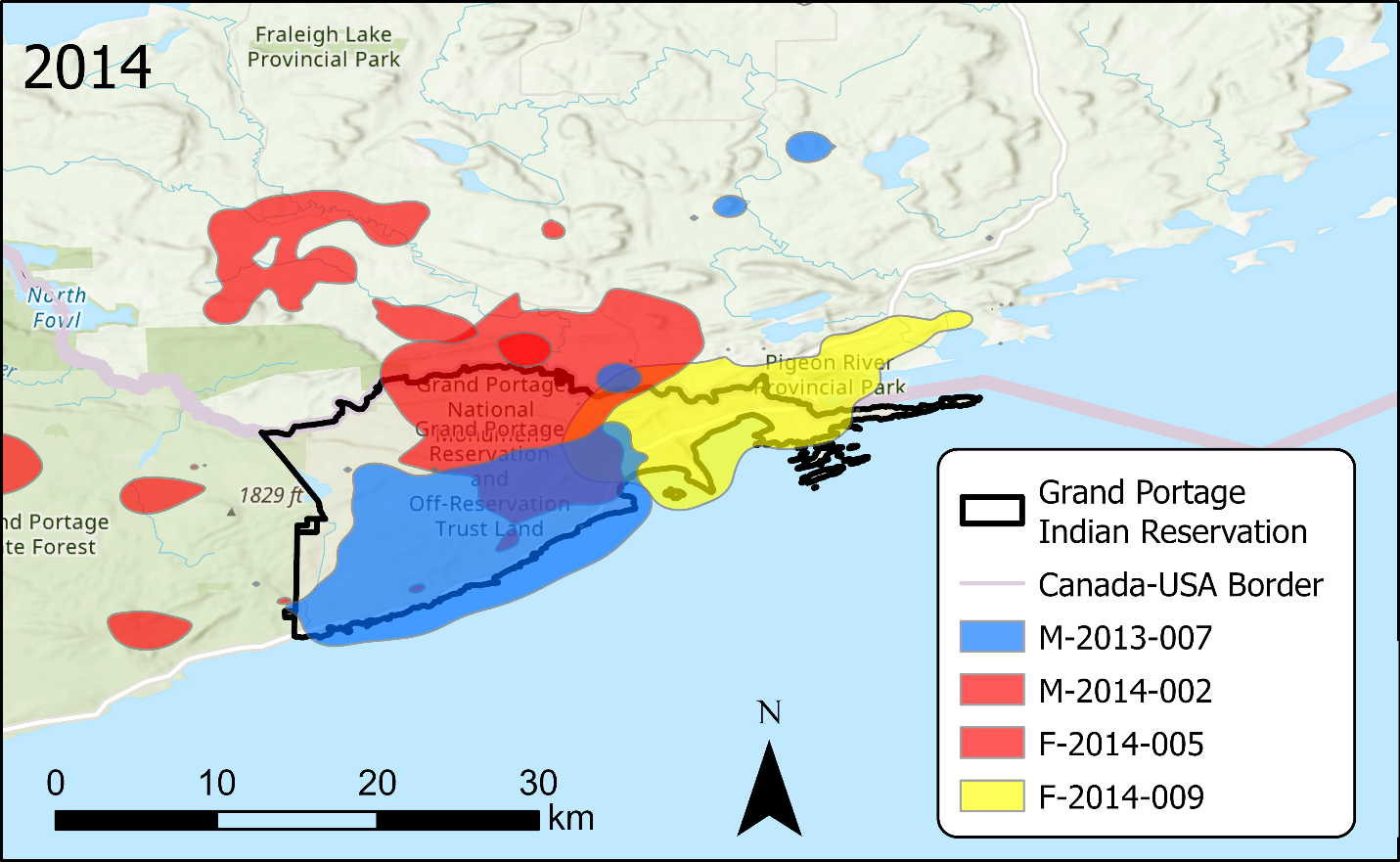
**

**
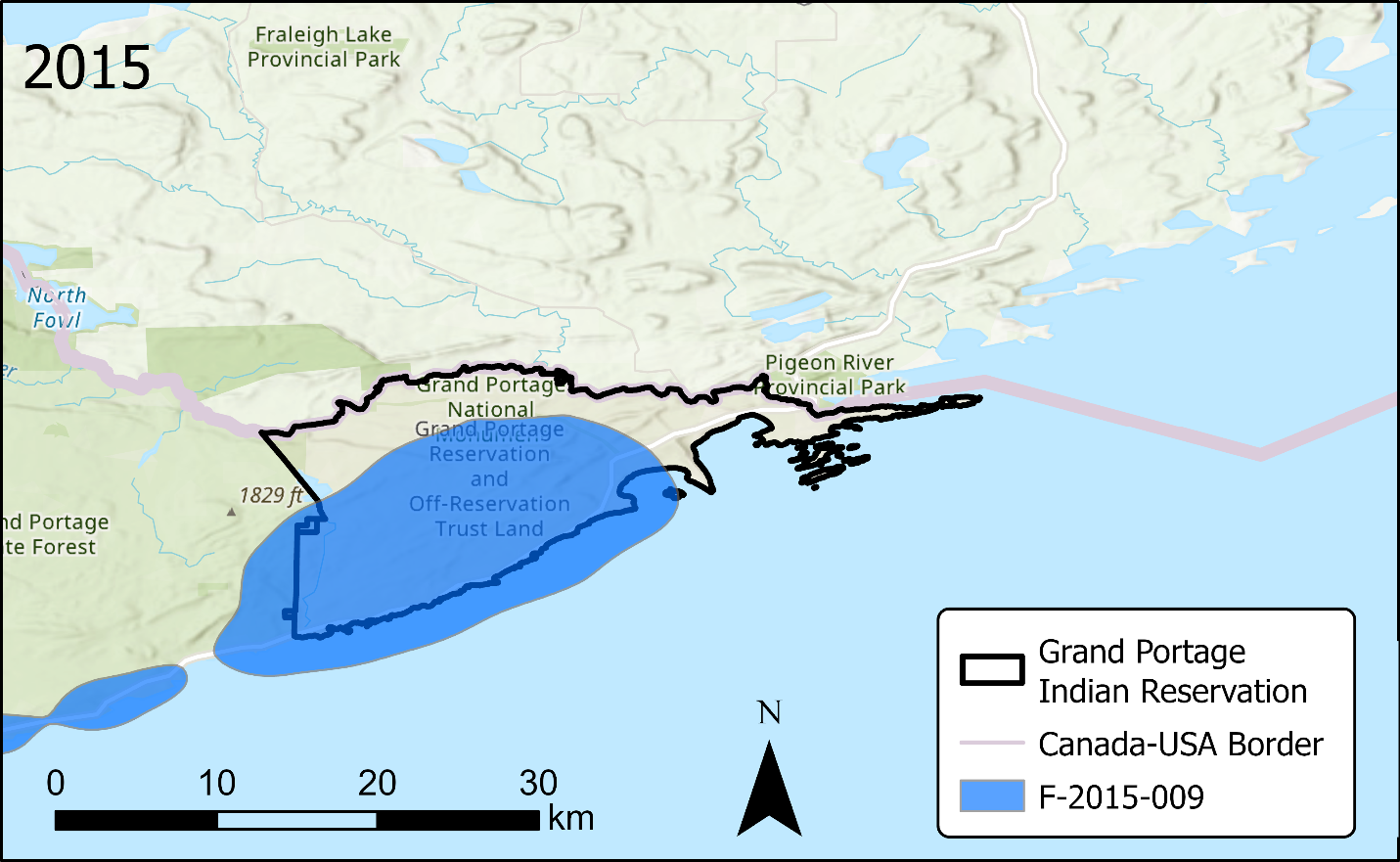
**

**Fig. S1** Continued

**
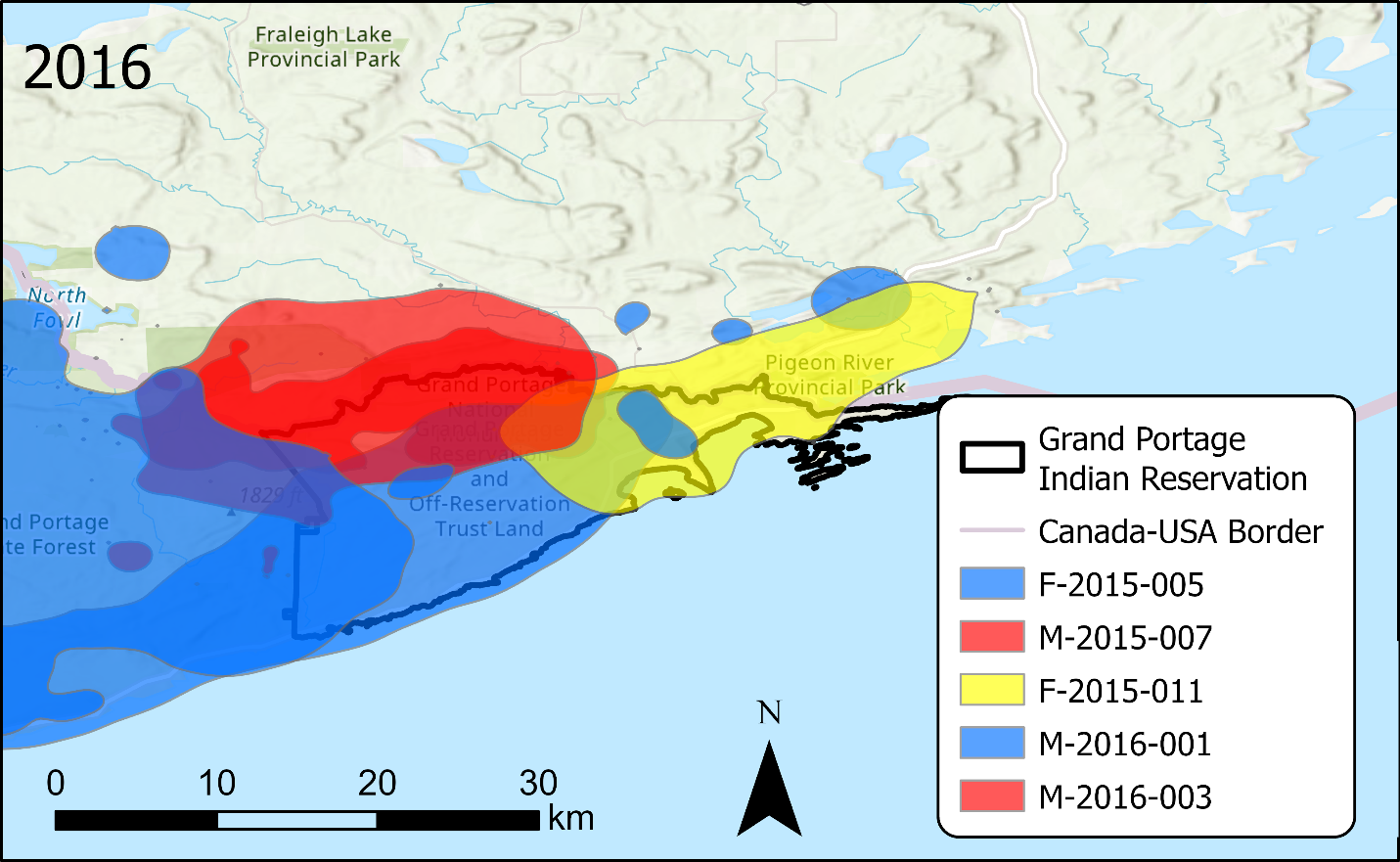
**


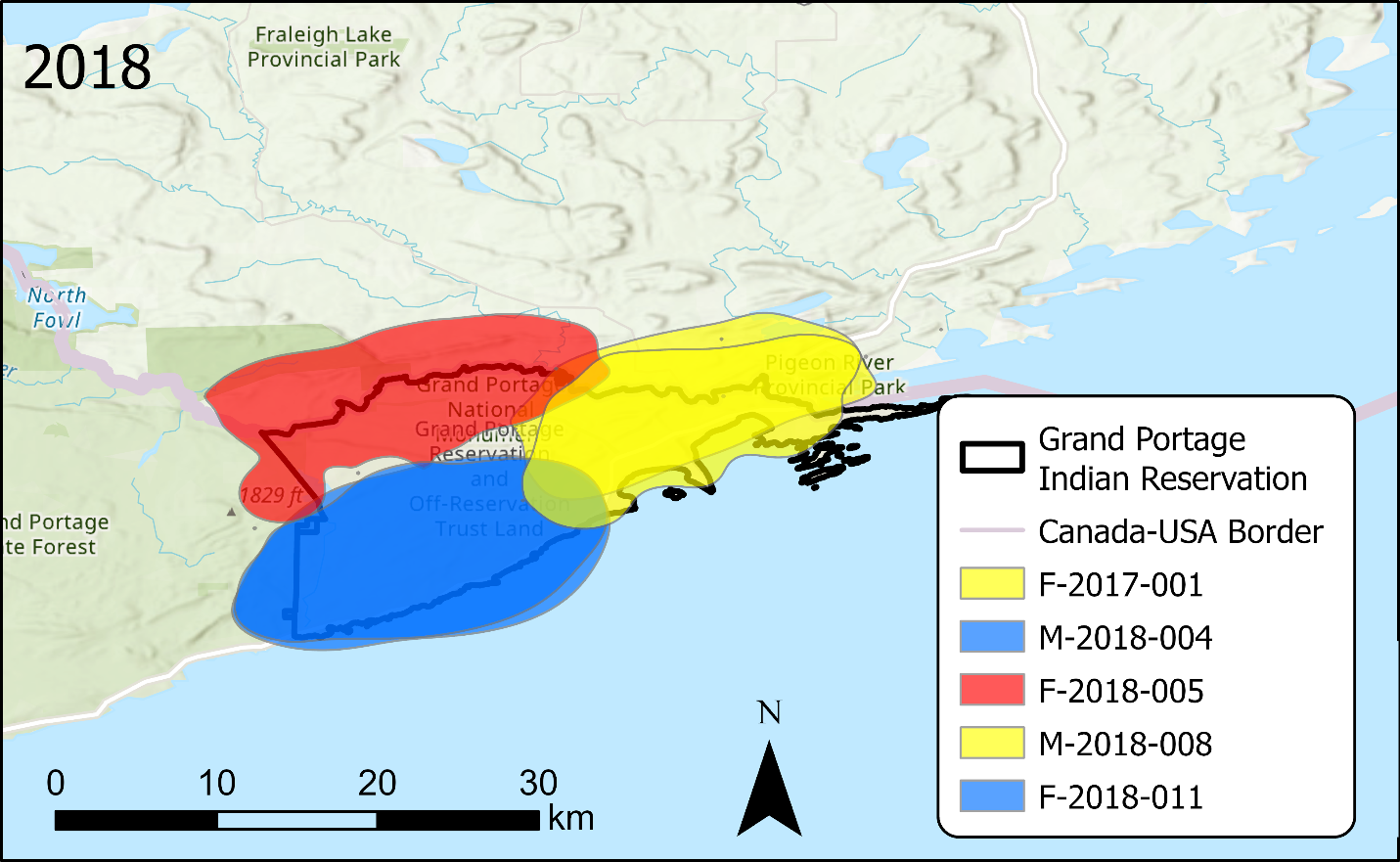


**Fig. S1** Continued


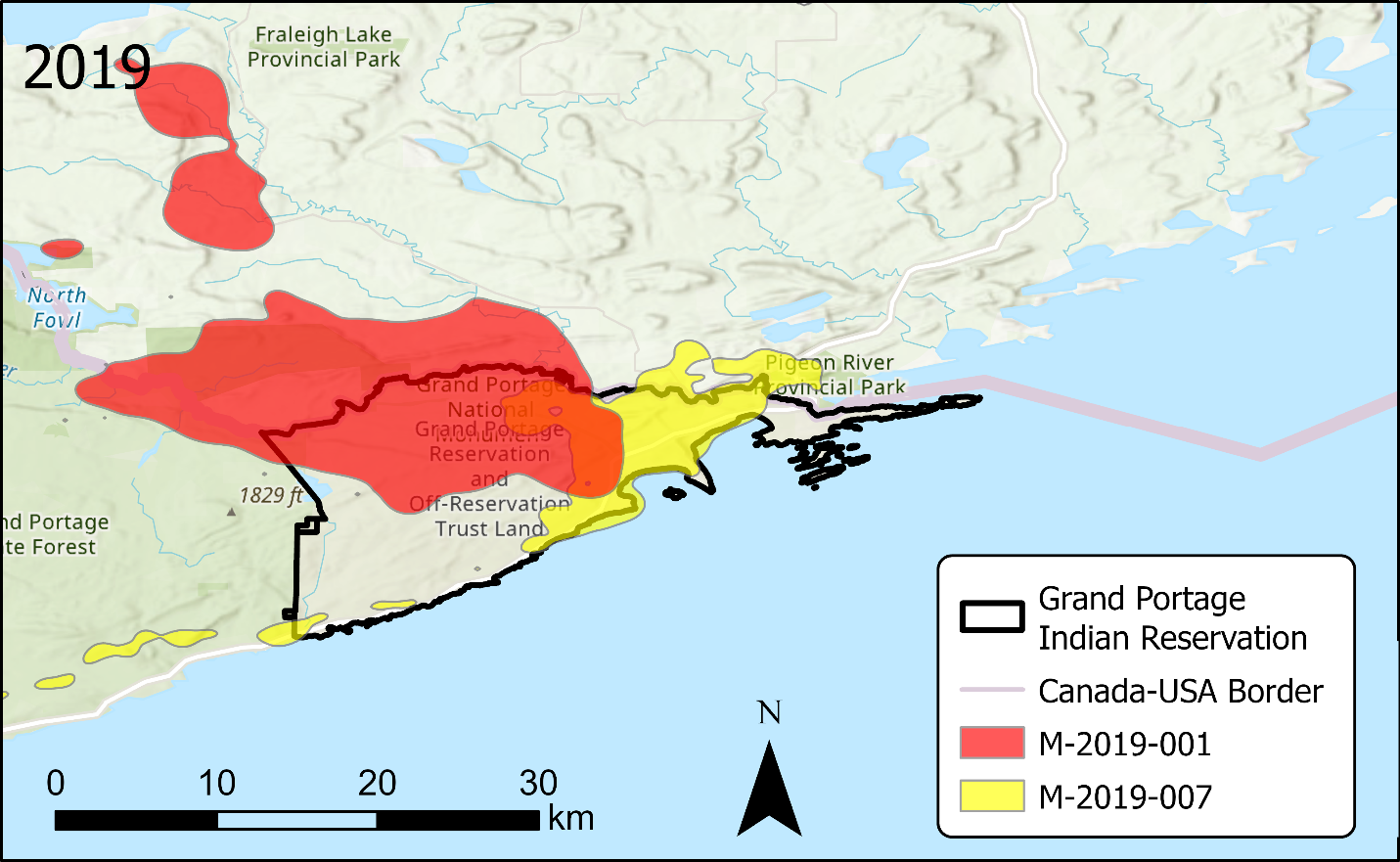


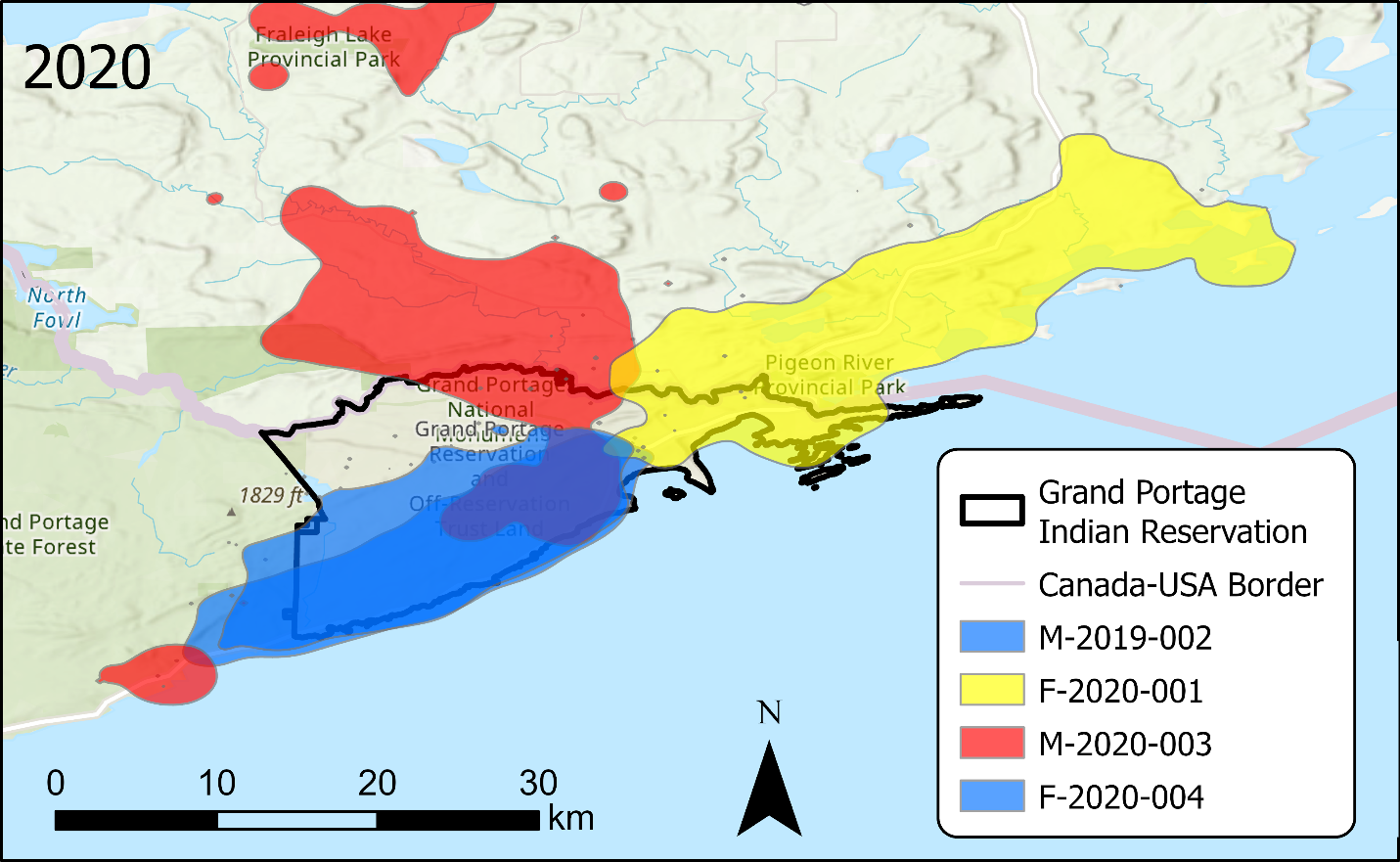


**Fig. S1** Continued


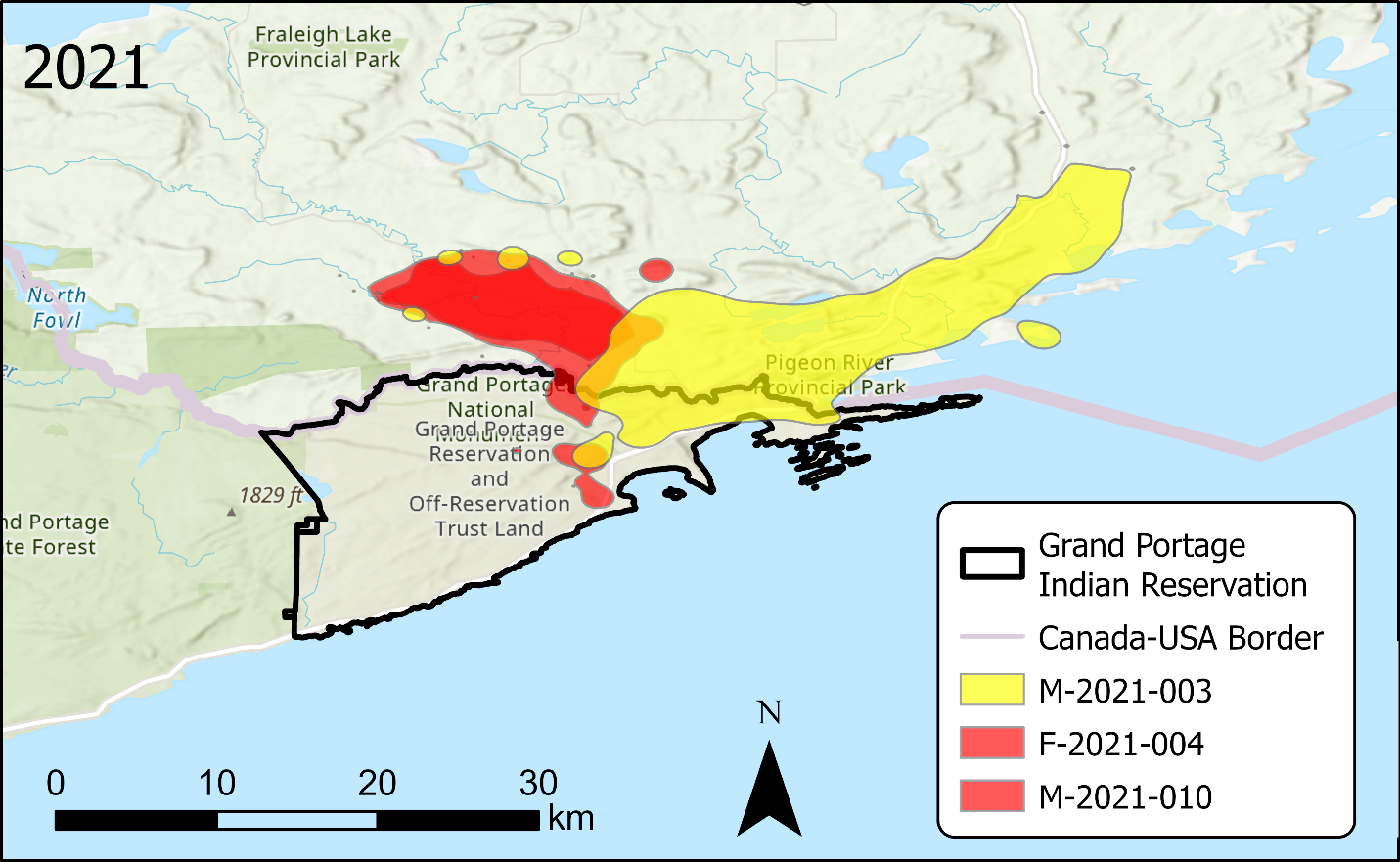


**Fig. S2** Monitoring periods of gray wolves (*Canis lupus*) on and near the Grand Portage Indian Reservation, Minnesota, USA, 2013–2021. Wolf identifiers on the y-axis include sex (M = male, F = female) and 7-digit individual ID; colors delineate pack affiliation (orange = floater [FL], yellow = northeast [NE], red = northwest [NW], blue = southwest [SW]). Date is on the x-axis. Most captures occurred during July–October.

**
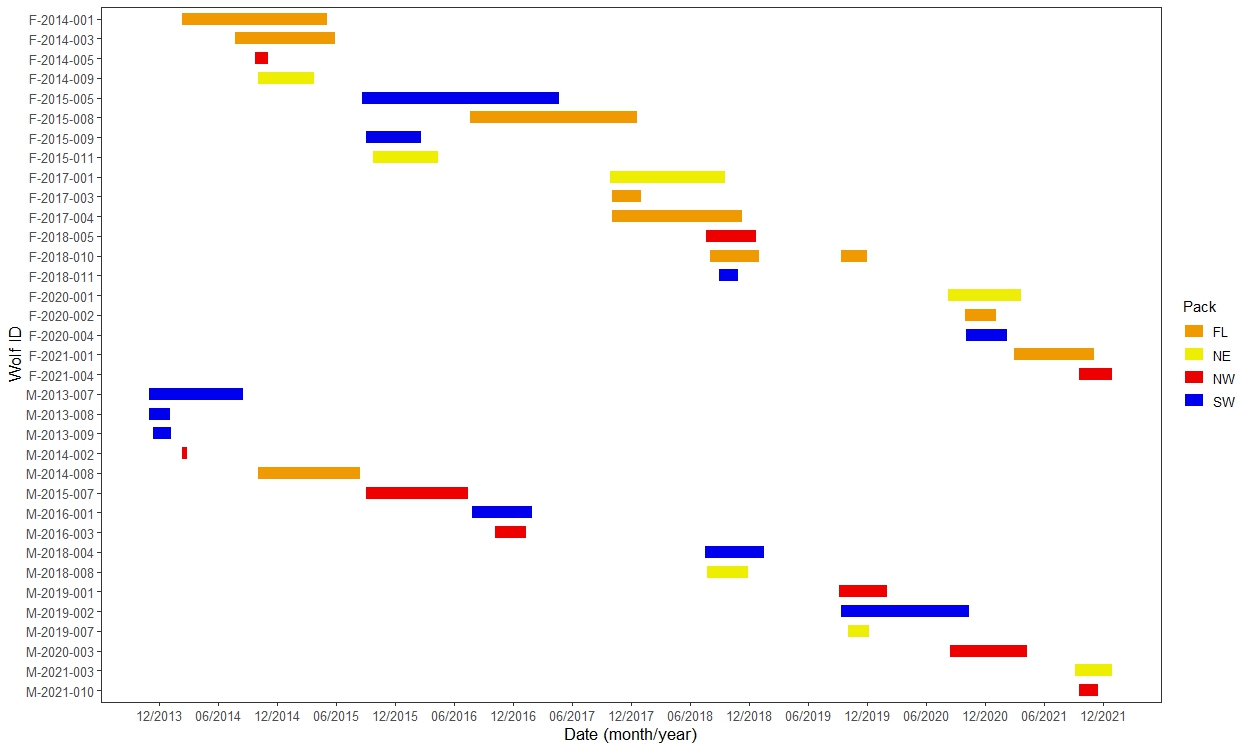
**

**Fig. S3** Still image of white-tailed deer (*Odocoileus virginianus*) spring migration on and near the Grand Portage Indian Reservation, Minnesota, USA, 2016–2021. Green dots represent migratory deer, blue dots represent resident deer, and red dots represent deer that dispersed or whose movement strategies could not be assessed. Black shading represents the combined spring and fall migration corridor. The year in chronology was altered to merge movements across years. The full video is available in a separate manuscript attachment.


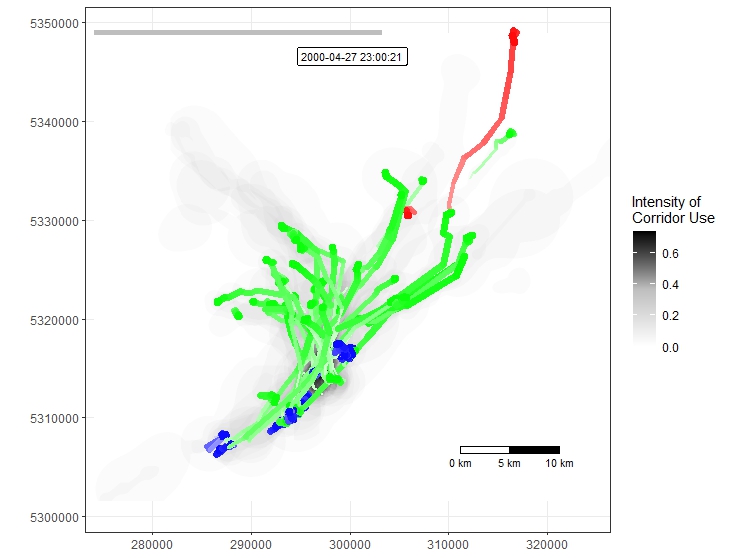


**Fig. S4** Still image of white-tailed deer (*Odocoileus virginianus*) fall migration on and near the Grand Portage Indian Reservation, Minnesota, USA, 2016–2021. Green dots represent migratory deer, blue dots represent resident deer, and red dots represent deer that dispersed or whose movement strategies could not be assessed. Black shading represents the combined spring and fall migration corridor. The year in chronology was altered to merge movements across years. The full video is available in a separate manuscript attachment.


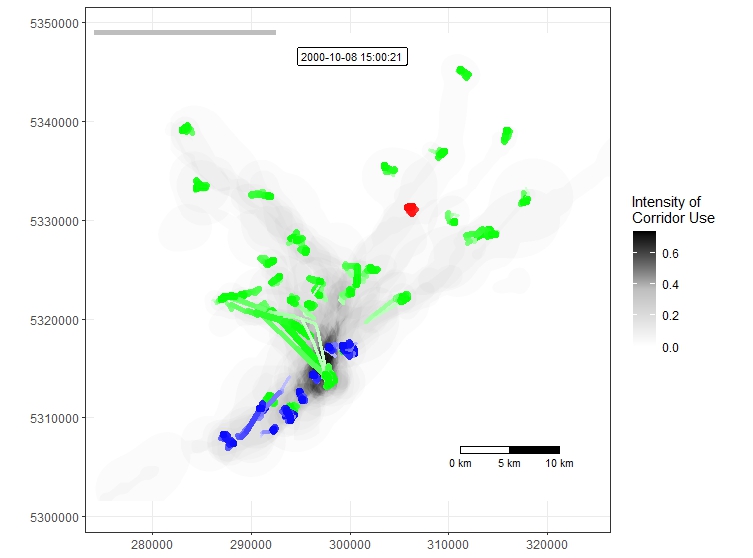


**Fig. S5** Seasonal proportional overlap of gray wolves (*Canis lupus*) with the white-tailed deer (*Odocoileus virginianus*) migration corridor on and near the Grand Portage Indian Reservation, Minnesota, USA, 2012–2021. Proportion of overlap was calculated using 95% wolf UDs and 95% occurrence distributions of migratory deer during their spring and fall migration events. Points represent proportion of overlap for each wolf monitored during winter, spring migration (spring), summer, and fall migration (fall). Individual wolf characteristics are indicated where circles represent females, triangles represent males, and colors indicate pack affiliations (FL = floating, NE = northeast, NW = northwest, and SW = southwest).


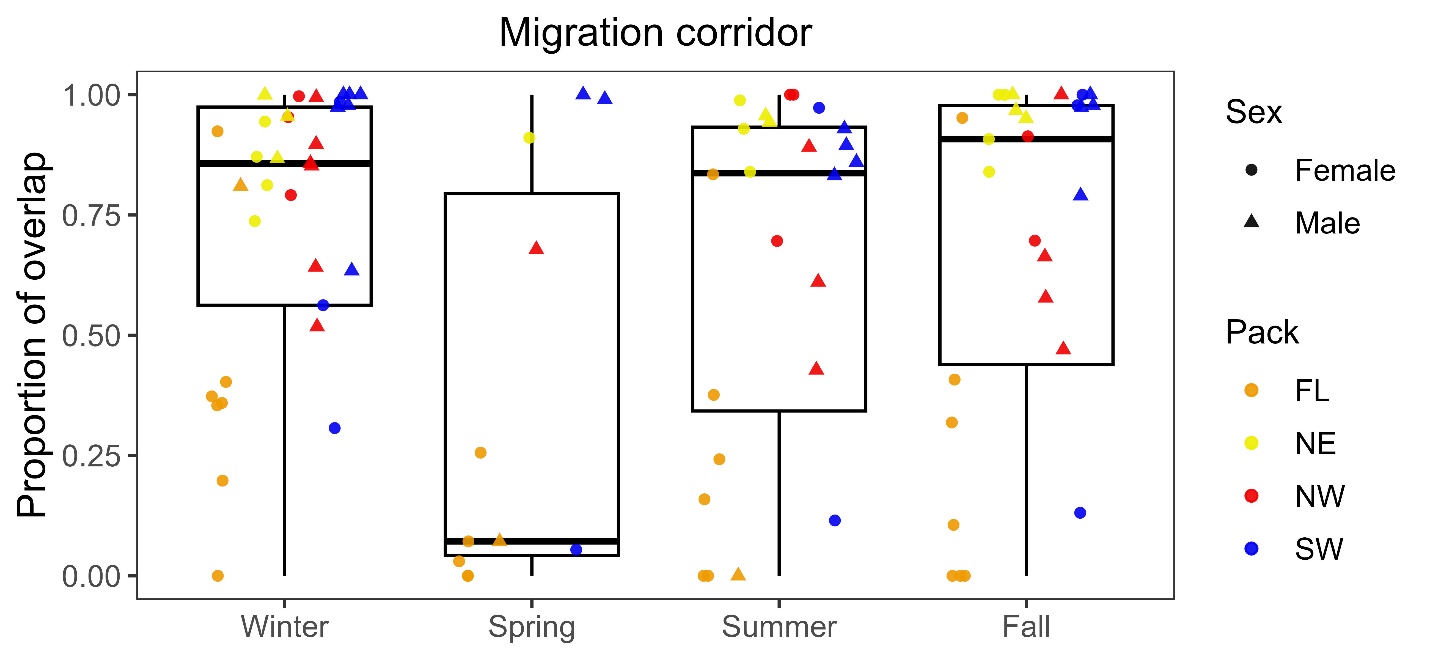

Supplement: Supplementary file 3 — Supplementary Material 3 [file 40462_2024_466_MOESM3_ESM.docx]
